# Supplementary material for: Heparin-based hydrogel scaffolding alters the transcriptomic profile and increases the chemoresistance of MDA-MB-231 triple-negative breast cancer cells
Source: Biomater Sci. 2020 Feb 13;8(10):2786–96. doi: 10.1039/c9bm01481k (PMC7497406; doi:10.1039/c9bm01481k)
Supplement: Supplementary file 2 [file BM-008-C9BM01481K-s002.zip › Supplementary File 4/HP-B hydrogel + EGF (s)vControl/Pathways/my_analysis.Gsea.1545201170987/HALLMARK_ANDROGEN_RESPONSE.html]

Details for gene set HALLMARK\_ANDROGEN\_RESPONSE[GSEA]

|  || Dataset | expr.class.cls#HYDEGF\_versus\_CONTROL.class.cls#HYDEGF\_versus\_CONTROL\_repos |
| Phenotype | class.cls#HYDEGF\_versus\_CONTROL\_repos |
| Upregulated in class | CONTROL |
| GeneSet | HALLMARK\_ANDROGEN\_RESPONSE |
| Enrichment Score (ES) | -0.51683193 |
| Normalized Enrichment Score (NES) | -2.225093 |
| Nominal p-value | 0.0 |
| FDR q-value | 0.0 |
| FWER p-Value | 0.0 |
Table: GSEA Results Summary

  

Fig 1: Enrichment plot: HALLMARK\_ANDROGEN\_RESPONSE      
 Profile of the Running ES Score & Positions of GeneSet Members on the Rank Ordered List

  

| PROBE | DESCRIPTION (from dataset) | GENE SYMBOL | GENE\_TITLE | RANK IN GENE LIST | RANK METRIC SCORE | RUNNING ES | CORE ENRICHMENT || 1 | HOMER2 | na |  |  | 236 | 2.509 | 0.0160 | No |
| 2 | RRP12 | na |  |  | 389 | 2.244 | 0.0334 | No |
| 3 | ACTN1 | na |  |  | 1089 | 1.628 | 0.0153 | No |
| 4 | PTPN21 | na |  |  | 1290 | 1.519 | 0.0220 | No |
| 5 | PMEPA1 | na |  |  | 1964 | 1.277 | 0.0012 | No |
| 6 | AKT1 | na |  |  | 2221 | 1.205 | 0.0014 | No |
| 7 | CCND1 | na |  |  | 2684 | 1.084 | -0.0105 | No |
| 8 | CDC14B | na |  |  | 2846 | 1.042 | -0.0071 | No |
| 9 | PTK2B | na |  |  | 3466 | 0.911 | -0.0292 | No |
| 10 | HERC3 | na |  |  | 3744 | 0.856 | -0.0341 | No |
| 11 | CENPN | na |  |  | 3769 | 0.851 | -0.0257 | No |
| 12 | DHCR24 | na |  |  | 4964 | 0.657 | -0.0807 | No |
| 13 | SRF | na |  |  | 6522 | 0.434 | -0.1573 | No |
| 14 | STEAP4 | na |  |  | 6684 | 0.413 | -0.1611 | No |
| 15 | UBE2I | na |  |  | 6745 | 0.404 | -0.1596 | No |
| 16 | CAMKK2 | na |  |  | 7149 | 0.352 | -0.1768 | No |
| 17 | UAP1 | na |  |  | 7199 | 0.344 | -0.1754 | No |
| 18 | CDK6 | na |  |  | 7387 | 0.319 | -0.1816 | No |
| 19 | STK39 | na |  |  | 7583 | 0.295 | -0.1885 | No |
| 20 | PIAS1 | na |  |  | 7777 | 0.266 | -0.1956 | No |
| 21 | FKBP5 | na |  |  | 7831 | 0.259 | -0.1954 | No |
| 22 | INPP4B | na |  |  | 8021 | 0.241 | -0.2026 | No |
| 23 | SGK1 | na |  |  | 8039 | 0.239 | -0.2008 | No |
| 24 | KRT8 | na |  |  | 8186 | 0.226 | -0.2059 | No |
| 25 | ADRM1 | na |  |  | 8420 | 0.199 | -0.2158 | No |
| 26 | MERTK | na |  |  | 8571 | 0.178 | -0.2216 | No |
| 27 | CCND3 | na |  |  | 8864 | 0.140 | -0.2353 | No |
| 28 | NGLY1 | na |  |  | 8927 | 0.131 | -0.2371 | No |
| 29 | AKAP12 | na |  |  | 10058 | 0.011 | -0.2961 | No |
| 30 | SLC26A2 | na |  |  | 10074 | 0.009 | -0.2968 | No |
| 31 | MAK | na |  |  | 10228 | -0.013 | -0.3047 | No |
| 32 | ABCC4 | na |  |  | 10239 | -0.015 | -0.3050 | No |
| 33 | MAP7 | na |  |  | 10826 | -0.082 | -0.3348 | No |
| 34 | SORD | na |  |  | 10833 | -0.083 | -0.3341 | No |
| 35 | BMPR1B | na |  |  | 11316 | -0.139 | -0.3578 | No |
| 36 | ADAMTS1 | na |  |  | 11745 | -0.195 | -0.3780 | No |
| 37 | HMGCS1 | na |  |  | 11862 | -0.213 | -0.3817 | No |
| 38 | XRCC6 | na |  |  | 12225 | -0.249 | -0.3978 | No |
| 39 | ZMIZ1 | na |  |  | 12259 | -0.254 | -0.3966 | No |
| 40 | H1F0 | na |  |  | 12323 | -0.265 | -0.3969 | No |
| 41 | ABHD2 | na |  |  | 13290 | -0.384 | -0.4432 | No |
| 42 | PA2G4 | na |  |  | 13412 | -0.405 | -0.4449 | No |
| 43 | APPBP2 | na |  |  | 13497 | -0.412 | -0.4446 | No |
| 44 | ANKH | na |  |  | 13596 | -0.424 | -0.4450 | No |
| 45 | VAPA | na |  |  | 14248 | -0.513 | -0.4732 | No |
| 46 | HMGCR | na |  |  | 14351 | -0.528 | -0.4726 | No |
| 47 | RAB4A | na |  |  | 14856 | -0.612 | -0.4921 | No |
| 48 | GPD1L | na |  |  | 14857 | -0.613 | -0.4851 | No |
| 49 | TNFAIP8 | na |  |  | 15172 | -0.664 | -0.4941 | No |
| 50 | SPCS3 | na |  |  | 15475 | -0.716 | -0.5018 | No |
| 51 | GNAI3 | na |  |  | 15764 | -0.777 | -0.5080 | Yes |
| 52 | ELK4 | na |  |  | 15874 | -0.796 | -0.5047 | Yes |
| 53 | ELL2 | na |  |  | 15882 | -0.798 | -0.4961 | Yes |
| 54 | PDLIM5 | na |  |  | 15884 | -0.799 | -0.4871 | Yes |
| 55 | NKX3-1 | na |  |  | 15945 | -0.809 | -0.4811 | Yes |
| 56 | SMS | na |  |  | 16045 | -0.831 | -0.4769 | Yes |
| 57 | TPD52 | na |  |  | 16281 | -0.893 | -0.4791 | Yes |
| 58 | ALDH1A3 | na |  |  | 16296 | -0.897 | -0.4697 | Yes |
| 59 | GSR | na |  |  | 16589 | -0.967 | -0.4740 | Yes |
| 60 | FADS1 | na |  |  | 16789 | -1.024 | -0.4729 | Yes |
| 61 | TSC22D1 | na |  |  | 16950 | -1.072 | -0.4691 | Yes |
| 62 | KRT19 | na |  |  | 17022 | -1.096 | -0.4604 | Yes |
| 63 | SPDEF | na |  |  | 17163 | -1.131 | -0.4550 | Yes |
| 64 | TMPRSS2 | na |  |  | 17192 | -1.140 | -0.4435 | Yes |
| 65 | INSIG1 | na |  |  | 17226 | -1.153 | -0.4322 | Yes |
| 66 | XRCC5 | na |  |  | 17325 | -1.192 | -0.4239 | Yes |
| 67 | ELOVL5 | na |  |  | 17447 | -1.235 | -0.4163 | Yes |
| 68 | DNAJB9 | na |  |  | 17463 | -1.242 | -0.4030 | Yes |
| 69 | SEC24D | na |  |  | 17563 | -1.283 | -0.3937 | Yes |
| 70 | NCOA4 | na |  |  | 17593 | -1.292 | -0.3806 | Yes |
| 71 | ZBTB10 | na |  |  | 17658 | -1.317 | -0.3690 | Yes |
| 72 | IQGAP2 | na |  |  | 17663 | -1.320 | -0.3543 | Yes |
| 73 | SRP19 | na |  |  | 17734 | -1.351 | -0.3427 | Yes |
| 74 | RPS6KA3 | na |  |  | 17760 | -1.363 | -0.3286 | Yes |
| 75 | ACSL3 | na |  |  | 17998 | -1.484 | -0.3242 | Yes |
| 76 | UBE2J1 | na |  |  | 18003 | -1.486 | -0.3076 | Yes |
| 77 | B4GALT1 | na |  |  | 18015 | -1.493 | -0.2913 | Yes |
| 78 | PGM3 | na |  |  | 18040 | -1.508 | -0.2755 | Yes |
| 79 | IDI1 | na |  |  | 18098 | -1.547 | -0.2610 | Yes |
| 80 | ARID5B | na |  |  | 18389 | -1.746 | -0.2565 | Yes |
| 81 | TMEM50A | na |  |  | 18623 | -1.949 | -0.2466 | Yes |
| 82 | LIFR | na |  |  | 18713 | -2.068 | -0.2279 | Yes |
| 83 | ITGAV | na |  |  | 18729 | -2.091 | -0.2050 | Yes |
| 84 | SLC38A2 | na |  |  | 18823 | -2.234 | -0.1846 | Yes |
| 85 | NDRG1 | na |  |  | 18853 | -2.277 | -0.1604 | Yes |
| 86 | SCD | na |  |  | 18875 | -2.317 | -0.1353 | Yes |
| 87 | LMAN1 | na |  |  | 18876 | -2.320 | -0.1091 | Yes |
| 88 | MYL12A | na |  |  | 18878 | -2.323 | -0.0829 | Yes |
| 89 | DBI | na |  |  | 18967 | -2.501 | -0.0592 | Yes |
| 90 | SAT1 | na |  |  | 19134 | -3.131 | -0.0325 | Yes |
| 91 | B2M | na |  |  | 19139 | -3.164 | 0.0031 | Yes |
Table: GSEA details [plain text format]

  

Fig 2: HALLMARK\_ANDROGEN\_RESPONSE      
 Blue-Pink O' Gram in the Space of the Analyzed GeneSet

  

Fig 3: HALLMARK\_ANDROGEN\_RESPONSE: Random ES distribution      
 Gene set null distribution of ES for **HALLMARK\_ANDROGEN\_RESPONSE**
